# War on the Vampire; a Layman in the Lead

**Published:** 1906-03

**Authors:** 


### TEXAS MEDICAL JOURNAL.

AUSTIN, TEXAS.

A MONTHLY JOURNAL OF MEDICINE AND SURGERY.

EDITED AND PUBLISHED BY
F. E. DANIEL, M. D.
Mrs. F. E. DANIEL, Business Manager.

Published Monthly at Austin, Texas. Subscription price $1.00 a year in advance

"A man who maintains a hopeful, confident, fearless mental attitude will attract to himself like thoughts from others, and will be strengthened and helped by the influence of the outside thought, and will go on, from success to success."—*The New Thought.*

### WAR ON THE VAMPIRE; A LAYMAN IN THE LEAD.

I reproduce in this issue a proposed bill to regulate the manufacture and sale of patent and proprietary medicines, gotten up by Mr. Bok, editor of *The Ladies' Home Journal,* and published in that paper. It is an excellent bill and, with certain modifications, should become a law; but it is too sweeping, in that it includes in its restrictions and prohibitions the well-known, tried and valuable trade-marked or proprietary articles that contain no poisons whatever; pharmaceuticals, for instance, like Listerine, Kasagra, Pepto-Mangan, Glycothymoline, Tongaline, and many others I could name. They are manufactured on a large scale for physicians' use and are advertised to physicians only. Take Hayden's Viburnum Compound. It is a prescription originating with and used by the late venerable Dr. W. R. Hayden, of Massachusetts, than whom a more ethical, able and in every way reputable physician never lived. The restrictions in the bill, in order to hit what Mr. Bok is hitting at (and which ought to be hit), and yet not harm the harmless and reputable preparations, should apply, and be confined, to patent, and proprietary, and *officinal* preparations of the U. S. P. that *contain poisons;* and in the list, in addition to the poisons enumerated by Mr. Bok, should be included carbolic acid, strychnia, arsenic, aconite, nux vomica, acetanelid, chloroform, etc. All mixtures containing any of these, in addition to morphin, chloral, cocaine, wood alcohol, etc., whether patented or proprietary or officinal in the U. S. D., should be labeled "poison." To compel the manufacturers of the non-toxic proprietaries intended for physicians' use only, to publish the formula of their product, would be as senseless as it would be unjust; for not only would it be equal to confiscating their property and driving their preparations out of every State, thus depriving those who wish to prescribe them, of the possibility of getting the genuine, but it would stimulate and increase the great evil of substitution; for nine-tenths of the retail druggists would attempt to imitate these popular remedies, and thus work an injury to the physician as well as the manufacturers.

This bill or a similar and better one, free from the defects pointed out above, and those pointed out by the *Journal of the American Medical Association* (reproduced herewith in part), should have originated with the Council of Pharmacy of the A. M. A., who have been, and are, working along this line; and it is little credit to the profession that a layman should have led off. It is proposed that this bill shall be introduced in all the State Legislatures simultaneously. A similar bill was introduced into thirteen legislatures last winter and defeated in every one. Why? I'll tell you why: The secular and religious press are almost, without an exception, supported by these advertisements, and are arrayed against reform and will fight it. There is too much money used to defeat such bills, and too many "honorables" ready to take it. One strong man in the

Senate, say, who has been *"fixed,"* can fix many, perhaps a majority, by refusing to support some of their pet and perhaps paid-for bills, unless they will vote with him, and to my certain knowledge Senators and "reps" *swap votes.*

The bill will not pass. Already at this writing a similar bill has been defeated in Mississippi and in other States.

The second clause should become a law, but it never will; or, if so, it will not be enforced (more's the pity), and the entire draft of the bill, if amended so as to exempt non-toxic preparations, and amended in other respects as pointed out, should become a law; but it never will (more's the pity). Already we have on our statute books in Texas a law that prohibits the sale of all poisons,—even paregoric,—except on physician's prescription; but they are sold all the same, to anybody, by all druggists,—the headache powders, and cocaine-snuff stuffs, and coryza tablets, etc. (morphin). In 1885 the Texas Legislature passed a sensible and most comprehensive law to regulate the sale of poisons and the adulteration of food, drugs, and drinks; but they failed to make any appropriation to back it, and refused to create a State Board of Health to execute it, clinging on like grim death to the "one-man band"—a single quarantine officer, improperly called "State Health Officer" (inasmuch as Texas takes no heed of anything affecting the public health except yellow fever and smallpox) and that excellent law is inoperative and a dead letter today and has ever been. That bribery will defeat all medical legislation that touches the pockets of those who are thriving upon the necessities, credulity and ignorance of the public can not be doubted. The same applies as well to the U. S. Senate as to State Senators. Read this from the *Cosmopolitan* for March, by David Graham Phillips:

> "'The Treason of the Senate!' Treason is a strong word, but not too strong, rather too weak, to characterize the situation in which the Senate is the eager, resourceful, indefatigable agent of interests as hostile to the American people as any invading army could be, and vastly more dangerous; interests that manipulate the prosperity produced by all, so that it heaps up riches for the few—interests whose growth and power can only mean the degradation of the people, of the educated into sycophants, of the masses toward serfdom.
>
> "The Senate, that is the Senators as Senators, are false to their oaths, false to the people; they are faithful, with assiduous fidelity of the pocket-interest.

Mr. Bok has expressed some surprise at the lukewarmness of the medical profession in this matter. He says the doctors do not go before the legislative committees, etc., and he reads them the riot act. The reason, or one of the reasons, why, has been stated above. Many physicians think the bill, as proposed, is unjust, too sweeping, and is not practicable.

There is another reason: We here in Texas have seen and felt the effect of bucking against hostile interests, and some of us are disgusted with the eternal wrangle over "pathies," "separate boards for separate schools," etc., and with the conduct of certain irascible, slippery and sentimental "senators"; and no self-respecting physician feels like subjecting himself to the humiliation of a repetition of last session's fiasco, snubbing and brutal insults, nor to face again certain defeat. The majority are not in sympathy with that optimism and spirit of sycophancy—and of turning the other cheek—that prompts to pocketing the affronts and hoping for "better luck next time." Unless the physicians take stock and become a factor in the next election, and help to relegate certain narrow and unreliable "friends" to the shades of private life, sending better, wiser and more trustworthy men in their places, we will never succeed in getting legislation.

In the matter of legislating against patent medicines, the hopelessness, under the circumstances, of bucking against a $250,000,-000 trust, backed by the great majority of newspapers, is paralyzing.

The indiscriminate sale and use of health-destroying and life-destroying medicines by the people is the crying evil of the day. It is The Great National Vampire, the most potent factor in the swift decay of the race. It is an incubus, fastened tighter than an "old man of the sea" upon the public, that nothing will shake off so long as there are purchasable "honorables" and barrels of money. More's the pity. May God have mercy on the victims of this great evil; for the medical men seem powerless to procure laws for their protection.

Our legislative committee will vigorously take up the matter of sanitary legislation in April; but—what's the use? They will not give us a Board of Health nor money enough even for quarantine.

A Physician's Prescription Pharmacy.—The passage of such a sweeping law as proposed by Mr. Bok would play into the hands of the substituting retail druggists, the doctors' worst enemies—the promoters and exploiters of the worst kind of patent medicines. The stock of the retail druggist is composed principally of the fraudulent and poisonous patent medicines, and when a person calls at a drug store for a doctor, the proprietor or clerk will recommend something for his "case" and sell him a cure-all. The prescription druggist is an anachronism. He is a fifth wheel and should be cut out. He should be boycotted who does these things; and every physician should dispense his own medicines; or, better still, I suggest that in every town of sufficient population the doctors combine and employ an honorable and capable man to fill their prescriptions, giving him a salary or a percentage. I suggest

A Physicians' Co-operative Pharmacy, a drug store where no fraudulent and poisonous patents are to be sold under any circumstances. Let all practitioners in that town send their prescriptions to this one place, and thus eliminate their principal enemy and competitor—the patent medicine selling and substituting druggist. A stock of perfumery, stationery, cigars, etc., could be carried, in addition to everything a doctor needs in his work. Think of it.

Invoke the Big Stick. With two hundred and fifty millions back of it, purchasable "honorables," and the secular and religious press practically a unit in its support, the Great American Fraud, as *Collier* calls it, snaps its fingers at us and says: "The public be damned." Witness the Pure Food bill, which has been before Congress *seventeen years,* and is still hanging fire before the present Congress. It will not pass. (P. S.—Much modified, it has passed the Senate.)

The only hope is to create a public sentiment by educating the masses—if that be possible—by showing how they are being victimized, and *Collier's* and the *Ladies' Home Journal* are doing excellent missionary work in this direction. This, with the elimination of the patent medicine selling and substituting druggist, is the only possible hope,—*until we get a stronger form of government* with power in the hands of the head, to do as they do in Mexico, for instance; but—

Speaking of Mexico, read this. Shame on Uncle Sam, that this people should have a better appreciation of sanitary science and power and determination to enforce it than we. Our pitiful appropriation in Texas is expended solely on quarantine, and is not enough for that:

Mexico City, Feb. 15.—The sanitary campaign to improve the health conditions of the poorer classes has already been waged with such vigor as to diminish the death rate from fevers. Public bathing places for the poor have been established all over the city and the superior board of health, which is backed by the Federal government, hopes to control the infant mortality and show excellent results within a few months.

---

TEXAS STANDS PAT, and in the matter of quarantine control by the U. S. Government reminds me of the juror who stood out for conviction against the eleven others for acquittal. He said they were the most obstinate fellows he ever met. At the quarantine convention at Chattanooga, of 14 Southern States, Texas alone was not represented. In caucus the Williams' bill, relegating control of quarantine to the U. S. Public Health and M. H. Service, in accordance with the resolution unanimously adopted at that convention, was agreed to by everybody—except the Texas congressmen. I am reminded of the story of the bull and the locomotive: "good pluck, but d—d poor judgment." The bill provides that the U. S. shall manage international quarantine and shall manage state and interstate quarantine jointly with the State authorities. Is that not just what is being done now? And every time we get in a tight place we have to hand over the reins and the cost to Uncle Sam. Is not a double line of quarantine kept up, both maritime and land—one by States and one by M. H. S.? What's the use? Texas was only too glad to get the M. H. S. to manage the smallpox outbreak of Eagle Pass in 1892 or 4. It cost $214,000. Where would our little appropriation have been? In 1882 the U. S. government had to come to the relief of Texas in the yellow fever epidemic near Brownsville, and also at Laredo in 1903. It makes me tired to hear talk of States' rights. I am on record as standing for States' rights in this matter, and I toted a gun in defense of it forty-odd years ago; but as we were whipped out then—"might" being "right"—we are compelled to surrender in the quarantine matter because conditions are changed and because Texas is too poor or too mean to assert her rights and go it alone. The shortage on account of quarantine last year is $25,000, and there'll be a kick when the wise ones are called upon to make good.

Let Uncle Sam protect the States from disease invasion, as he would do from any other invasion, and pay the cost of it. The little forty-five or fifty thousand spent on Texas quarantine could then be used to remove numerous causes of death — other than yellow fever and smallpox. We have not a cent for the improvement of sanitary conditions and the prevention of preventable diseases that destroy a hundred times as many lives as yellow fever and smallpox. I surrender States' rights.

---

THE BARS ARE DOWN; all schools are admitted to the A. M. A. under the Simmons regime. I take the following from the *Medical Sentinel,* Portland, Oregon:

"Dr. J. N. McCormack's letter in the journal of the American Medical Association did the medical profession of Oregon a grave injustice. Yet, so far as Portland is concerned, it served at least one useful purpose, in calling attention to the difference that exists between the entrance requirements of the National Association and those of the city and county society. The former body declares that any practitioner of medicine who is a gentleman, *regardless of school of practice,* may become a member. The portals of admission are through the State societies, which, in turn, are made up of the members of the various county and district bodies. Thus membership in the National body, which officially represents the entire profession, is a matter definitely determined by the local official societies. * * * It therefore appears that, far from dictating who shall and who shall not become a member of the local society, the American Medical Association has delegated to that body the privilege of naming those who may or may not become members of the parent organization, *stipulating only that no discrimination shall be exercised against candidates merely because schools of practice differ.*" [Italics are mine.—DANIEL.]

Well, well, well. Let slip the dogs of war and cry "havoc," and all go in for the make. "Every little bit helps"; that is, a $5 subscription to the Octopus is as good from an osteopath, a homo. or a faith-healer as from a "regular." The A. M. A. has become an *omnum gatherum* and is no longer either fish, flesh or fowl, nor yet good red herring, but a mixture of them all. The old Code recited: "Their sectarian title makes them quacks." Shades of Davis and Hamilton, go weep. Will the reputable and self-respecting physician be thus like dumb cattle driven and made to herd with all kinds of pathies? More than one of my subscribers has written me that it lets him out; one says, after a membership of thirty years.

